# The Impact of Blue-Green Space Landscape Patterns on Bird Richness in Southwest China

**DOI:** 10.3390/ani16121792

**Published:** 2026-06-10

**Authors:** Xingru He, Siyuan Li, Ziling He, Qinmei Yan, Jingwei Shen

**Affiliations:** 1 Chongqing Engineering Research Center for Remote Sensing Big Data Application, Chongqing Jinfo Mountain Karst Ecosystem National Observation and Research Station, School of Geographical Sciences, Southwest University, Chongqing 400715, China; he112025318001267@email.swu.edu.cn (X.H.); lsy020379@email.swu.edu.cn (S.L.); heziling@email.swu.edu.cn (Z.H.); yqm6473223@email.swu.edu.cn (Q.Y.); 2 Key Laboratory of Monitoring, Evaluation and Early Warning of Territorial Spatial Planning Implementation, Ministry of Natural Resources, Chongqing 401147, China; 3 Research Center for New Land-Sea Corridors and Regional Development, Southwest University, Chongqing 400715, China

**Keywords:** blue-green space landscape pattern, bird richness, RF model, SHAP analysis

## Abstract

With the accelerated pace of urbanization, the substantial reduction in natural vegetation, water bodies, and wetlands has disrupted ecosystem structures, leading to significant declines in biodiversity. Blue-green spaces play a crucial role in maintaining urban habitat quality and supporting species diversity. As a sensitive indicator group to changes in the ecological environment, spatial variations in bird richness can provide important insights into changes in urban ecosystems and habitats. Therefore, a systematic investigation of the relationship between the landscape patterns of blue-green spaces and bird richness in ecologically complex regions is of great significance for achieving sustainable urban development and biodiversity conservation. This study focuses on Southwest China, utilizing bird richness data and blue-green space landscape pattern indicators. By integrating Random Forest (RF) models with Shapley (SHAP) methods, it quantitatively analyzes the relationship between blue-green space landscape patterns and bird richness in typical complex ecological regions. Results indicate nonlinear associations between blue-green landscape patterns and bird richness, with green spaces exerting a stronger overall influence than blue spaces. Edge density (ED) in green spaces demonstrated markedly higher feature importance than other landscape indicators. Within green spaces, ED and class area (CA) showed stronger associations with bird richness, while within blue spaces, CA and Percentage of Landscape (PLAND) provided more prominent explanatory power for bird richness. By clarifying the nonlinear responses and differentiated roles of blue and green landscape patterns, this study provides quantitative evidence for optimizing blue-green spatial planning and promoting biodiversity conservation in ecologically complex regions.

## Simple Summary

Rapid urban growth is reducing natural vegetation, water bodies, and wetlands, which harms ecosystems and lowers biodiversity. A key challenge is how to design urban blue and green spaces, such as parks and rivers, to improve environmental quality. This study focuses on Southwest China and examines how the layout of these spaces affects bird diversity, an important sign of ecological health. The results indicate that the relationships are complex and nonlinear. Green spaces have a stronger overall influence than water areas, and the edges and size of green spaces are especially important. The findings suggest that better planning of parks and water systems can improve urban environments and support more wildlife. This provides clear guidance for building healthier and more sustainable cities.

## 1. Introduction

With the accelerating pace of urbanization, land use patterns and ecological spatial structures have undergone significant changes, subjecting natural ecosystems to intense human disturbance [1,2]. Urban expansion often comes at the expense of existing natural vegetation, water bodies, and wetlands, leading to degraded habitat quality, environmental fragmentation, reduced ecological connectivity, and diminished ecosystem functions [3,4,5]. Against this backdrop, restoring and maintaining ecosystem health while enhancing biodiversity levels has become a core issue for global sustainable development [6,7].

Blue-green spaces serve as a vital ecological network, functioning both as a key component of regional natural environments that reflect multifaceted ecological conditions and social characteristics [8,9], and playing a crucial role in mitigating ecological degradation and maintaining biodiversity [10]. Leveraging blue-green space patterns as vital natural carriers is indispensable for sustaining ecological processes, providing diverse habitats, and promoting species diversity conservation [11]. Therefore, exploring how to enhance ecological functions and biodiversity by optimizing blue-green space patterns, grounded in the interrelationship between ecosystems and biodiversity, holds profound significance for regional ecological security and human well-being. As one of the most representative biological groups in ecosystems, birds exhibit high sensitivity to environmental changes and are widely used as ecological indicators for evaluating habitat conditions and biodiversity patterns at regional scales [12,13]. Therefore, to mitigate the negative impacts of future urban development on biodiversity, it is essential to investigate the associations of blue-green landscape patterns on bird richness and their underlying mechanisms.

Currently, avian research primarily focuses on four areas: (1) Fundamental studies on avian species diversity. This research concentrates on bird richness, diversity indices, community structure, and ecological behaviors. This foundational work provides insights into the survival status of birds in natural or artificial environments, quantifying their responses to environmental changes [14,15]. (2) Studies on the effects of different habitats and habitat characteristics on birds. This research emphasizes how habitat type, habitat structure, habitat fragmentation and connectivity, and habitat quality influence bird community composition, behavior, and functional diversity [16,17,18]. (3) Research on human impacts on birds. This category encompasses various direct and indirect human activities affecting birds, such as urbanization, human disturbance, domestication, climate change, artificial structures, and pollution [19,20]. (4) Research on bird conservation and ecological restoration. This research focuses on mitigating the negative impacts of human activities and urbanization through conservation actions, ecological restoration, and planning strategies [21,22]. Existing research on the relationship between bird diversity and landscape patterns has predominantly focused on micro- or city-scale settings such as urban parks, wetlands, or isolated green spaces [23]. These studies often rely on single ecological factors or traditional linear assumptions, making it difficult to reveal the overall response characteristics of bird richness under the synergistic effects of multiple blue-green space types in complex topographic regions. However, ecological responses of bird communities to landscape characteristics are often inherently nonlinear. Variations in habitat area, fragmentation, connectivity, and environmental heterogeneity may produce threshold effects, saturation effects, or abrupt ecological transitions [24,25]. For example, moderate habitat heterogeneity may increase ecological niche diversity and promote bird richness, whereas excessive fragmentation may reduce habitat stability and connectivity [26]. Similarly, increases in blue-green space area may initially enhance habitat availability, but their ecological benefits may gradually weaken once ecological resources approach saturation [27]. Traditional statistical methods therefore exhibit limitations in capturing these complex nonlinear relationships and threshold-dependent responses among high-dimensional landscape indicators, resulting in insufficient explanatory power regarding the ecological mechanisms linking blue-green landscape patterns and bird richness.

In recent years, advances in machine learning have opened up new research opportunities for uncovering the complex ecological relationships between landscape patterns and biodiversity. Among various machine learning models, the Random Forest (RF) model has been widely applied to species distribution prediction [28,29], habitat suitability assessments [30,31], and biodiversity research [32,33]. For example, previous studies have utilized RF models to assess the impact of three-dimensional urban park features on bird richness and to identify key environmental variables and nonlinear relationships influencing species richness in heterogeneous landscape settings [34]. Compared to traditional linear statistical models, RF is better able to capture threshold effects, nonlinear responses, and complex ecological interactions that are prevalent in urban and regional ecosystems. However, despite RF’s high predictive accuracy, its “black-box model” nature limits its ability to explain ecological mechanisms to some extent. To address this issue, the SHapley Additive exPlanations (SHAP) method has been increasingly adopted in ecological research to quantify the marginal contributions of individual variables [35,36]. By integrating RF with the SHAP framework, we can elucidate the mechanisms underlying species richness within landscape patterns.

Given the advantages of the aforementioned method, this study focuses on China’s ecologically complex southwestern region (Sichuan, Chongqing, Yunnan, and Guizhou provinces). By combining a random forest model with the SHAP interpretation framework, we investigate the nonlinear relationship between blue-green space landscape patterns and bird richness, quantify the contribution of different landscape indicators, and identify key response thresholds to better understand the ecological response patterns of bird richness in complex landscapes. The research aims to: (1) Reveal high-value and low-value zones for bird distribution clusters in Southwest China; (2) Further analyze the heterogeneity of blue-green landscape characteristics across different aggregation zones; (3) Utilize machine learning to assess the nonlinear relationship between bird richness and blue-green landscape patterns, along with key threshold ranges; (4) Propose corresponding bird conservation recommendations and landscape feature improvement strategies based on spatial landscape characteristics.

## 2. Materials and Methods

### 2.1. Study Area

**Figure 1:**
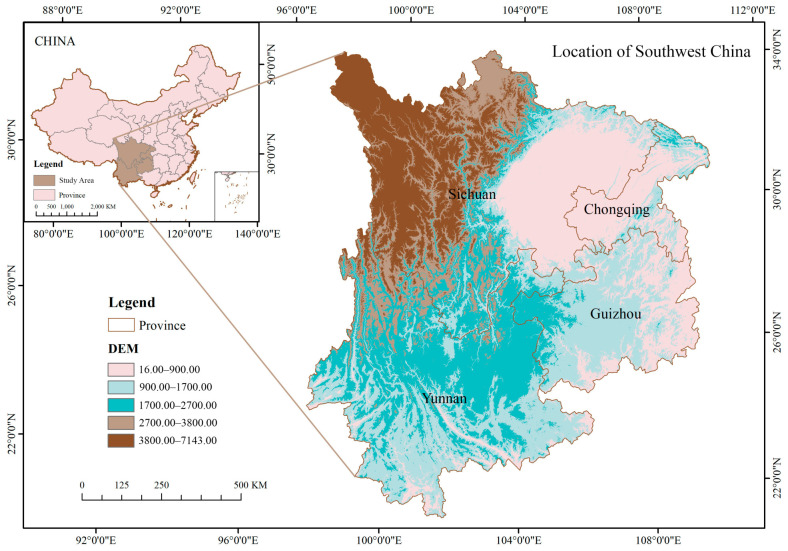
Location of Southwest China.

Southwest China is one of the country’s seven major geographical regions. This study selected Chongqing Municipality, Sichuan Province, Yunnan Province, and Guizhou Province (21°14′–34°31′ N, 97°35′–110°20′ E) within this region as the research area (Figure 1). The study area spans approximately 1.138 million square kilometers, with elevations ranging from 245 to 4497 m. Its permanent population stands at around 200.92 million (as of the end of 2023). The region comprises 438 districts and counties: 38 in Chongqing Municipality, 183 in Sichuan Province, 88 in Guizhou Province, and 129 in Yunnan Province. Economic conditions across the Southwest region exhibit significant disparities. Sichuan Province’s GDP totals approximately 6.47 trillion yuan, Chongqing Municipality’s GDP is about 3.22 trillion yuan, with per capita GDP exceeding 100,000 yuan, Yunnan Province’s GDP is approximately 2.90 trillion yuan, and Guizhou Province’s GDP is roughly 2.27 trillion yuan. Southwest China features predominantly mountainous terrain with limited flatlands, characterized by an intricate interweaving of basins, plateaus, gorges, and karst landscapes, resulting in complex and diverse topography. Annual average temperatures and precipitation vary significantly across counties and districts. For instance, Shiqu County in Sichuan Province has an annual average temperature of −3.1 °C, while Mengla County in Yunnan Province reaches 21.8 °C. The region exhibits diverse climatic patterns, including subtropical humid, temperate monsoon, and low-latitude plateau monsoon climates. The region is rich in rivers, forests, and grasslands, featuring extensive high-altitude mountain areas, grasslands, and evergreen forests and pastures. Yunnan Province, renowned as China’s most biodiverse province and often called the “Kingdom of Flora and Fauna,” possesses exceptional natural ecological resources. These provide ideal habitats for birds and serve as crucial stopover sites along the East Asian-Australasian Flyway.

### 2.2. Data Sources

This study utilized bird observation data from the China Birding Records Center, China’s largest citizen science bird database, including all bird records collected by the platform between 2014 and 2025. Bird richness was defined as the cumulative number of bird species recorded in each district and county during the study period. In addition, as the bird dataset was derived from citizen-science observations, the spatial distribution of records may be influenced by accessibility, observer activity, and uneven sampling intensity among regions. To reduce the influence of extreme observation gaps, bird richness was analyzed at the county scale, which may partially alleviate local-scale sampling fluctuations. Nevertheless, potential observation bias and detectability differences may still affect the estimated richness patterns. Therefore, the results should be interpreted as large-scale spatial association patterns rather than precise estimations of local bird community conditions.

The blue-green space data employed in this study were extracted from the annual China Land Cover Dataset (CLCD) with 30 m resolution, developed by Professor Huang Xin’s team at Wuhan University using Google Earth Engine. This dataset encompasses land use categories including forests, shrubs, water bodies, and wetlands. The Digital Elevation Model (DEM) data were sourced from the SRTMDEM raw elevation data available in the Geospatial Data Cloud. Other geospatial data, including precipitation and temperature, were derived from the ERA5-Land Daily Aggregated dataset to calculate annual average precipitation and annual average temperature. Normalized Difference Vegetation Index (NDVI) data were derived from the MOD13A3 product provided by the Moderate Resolution Imaging Spectroradiometer (MODIS; National Aeronautics and Space Administration, Washington, DC, USA). Built-up intensity (BUI) was obtained from the GHSL Built-up Surface (GHS-BUILT-S) released by the European Commission Joint Research Centre (JRC) [37]. BUI was used to represent urbanization intensity and anthropogenic disturbance (Table 1).

### 2.3. Methods

#### 2.3.1. Study Framework

**Figure 2:**
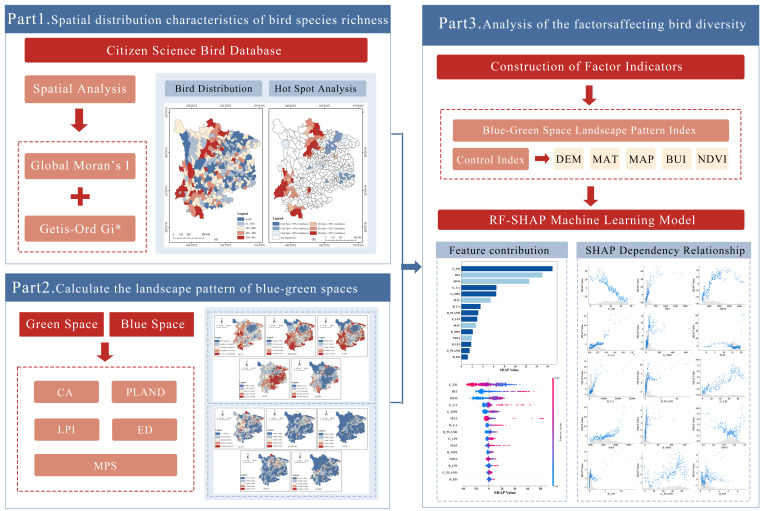
Research Framework. (1) Spatial pattern analysis of bird species richness using citizen science data and spatial statistics (Global Moran’s I, Getis-Ord Gi*). (2) Quantification of blue-green space landscape patterns via multiple indices. (3) Driving factor analysis using the RF-SHAP model to identify key factors affecting bird richness.

This study consists of three main parts. The first part analyzes the spatial distribution of bird richness and identifies areas with high and low bird aggregation within the study region using a citizen science bird database. The second part calculates landscape pattern indices for green spaces and blue spaces using six selected landscape pattern indicators. Finally, the Random Forest (RF) model and SHAP are employed to investigate the specific associations and threshold ranges of different blue-green space landscape patterns on bird richness. The research framework is illustrated in Figure 2.

#### 2.3.2. Quantification and Extraction of Landscape Features

Landscape pattern indices reflect the structural composition and spatial configuration characteristics of blue-green spaces, with relevant quantitative metrics computable using FRAGSTATS 4.2 software [38]. To better determine which types of ecological spaces drive changes in bird richness, we classified forest and shrub types from land-use data as green spaces, and categorized water bodies and wetlands as blue spaces [39]. This approach enables separate extraction and quantification of landscape patterns for subsequent independent driver analysis. This study selected six representative landscape pattern indices: Class Area (G_CA, B_CA), Edge Density (G_ED, B_ED), Largest Patch Index (G_LPI, B_LPI), Percentage of Landscape (G_PLAND, B_PLAND), and Mean Patch Size (G_MPS, B_MPS) (Table 2).

#### 2.3.3. Random Forest Algorithm and SHAP Model

Given the high dimensionality of landscape pattern variables and the potentially nonlinear relationships among environmental predictors, traditional statistical approaches may have limited ability to characterize complex ecological associations between blue-green space characteristics and bird richness. Therefore, this study employed a Random Forest (RF) model to explore the relationships between landscape variables and bird richness [28,29].

To improve model robustness and reduce overfitting, hyperparameter tuning was conducted using RandomizedSearchCV with five-fold cross-validation. The dataset was randomly divided into training and testing subsets at a ratio of 8:2. Model performance was evaluated using the coefficient of determination (R^2^) and root mean square error (RMSE) on the testing dataset.

To further interpret the contributions of individual variables, SHAP (SHapley Additive exPlanations) was applied. SHAP is a game theory-based interpretation framework that quantifies the marginal contribution of each predictor to model outputs, thereby enabling interpretation of nonlinear relationships and threshold responses between environmental variables and bird richness [35,36].

The basic SHAP formula is:

(1) $$\emptyset_{i}\left(f,x\right)=\sum_{S\subseteq F\backslash \left\{i\right\}}\frac{\left|S\right|!\left(M-\left|S\right|-1\right)!}{M!}\left[f_{S\cup \left\{i\right\}}\left(x_{S\cup \left\{i\right\}}\right)-f_{S}\left(x_{S}\right)\right]$$

Here, *F* denotes the entire feature set; $M=\left|F\right|$ represents the total number of features; *S* denotes the feature subset excluding feature i; $f_{S}\left(x_{S}\right)$ denotes the prediction value of the model trained using only feature subset *S* on sample *x*; the coefficient $\frac{\left|S\right|!\left(M-\left|S\right|-1\right)!}{M!}$ represents the probability of all permutations of feature entry sequences.

The additive interpretation formula for SHAP is:

(2) $$f\left(x\right)=\emptyset_{0}+\sum_{i=1}^{M}\emptyset_{i}$$

Among these, $\emptyset_{0}$ is the baseline value, typically the average predicted value across all samples in the training set; $\emptyset_{i}$ represents the contribution of feature i to the prediction (Shapley value).

## 3. Results

### 3.1. Spatial Distribution of Bird Richness

**Figure 3:**
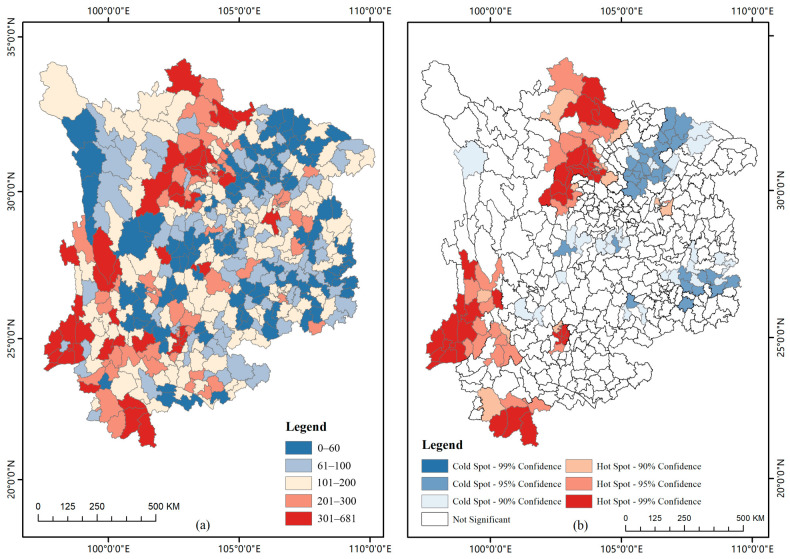
Bird Richness. (**a**) Graduated map of bird species richness across administrative units. (**b**) Hot spot (red) and cold spot (blue) analysis results based on the Getis-Ord Gi* statistic, showing significant spatial clusters at different confidence levels.

From 2014 to 2025, bird richness in different counties within the study area ranged from 0 to 681 species, as shown in Figure 3a. The areas with higher richness were mainly concentrated in the northern part of Sichuan Province and the western part of Yunnan Province. Overall, bird richness exhibited a spatial gradient characterized by relatively higher values in western regions and lower values in eastern regions.

The spatial autocorrelation analysis of bird richness indicates that it shows a positive spatial correlation (Moran’s I = 0.4), with a Z score of 13.95, suggesting that bird richness in the study area exhibits a spatial clustering trend rather than random dispersion. Therefore, the results of this study should primarily be interpreted as regional-scale ecological association patterns rather than strict causal ecological relationships.

To further determine the spatial heterogeneity and its specific locations, we conducted a cold-hotspot analysis (Figure 3b). The high-value spatial clusters are concentrated in the northern part of Sichuan Province and the western part of Yunnan Province, while the low-value spatial clusters are concentrated in the eastern part of Sichuan Province and the central part of Guizhou Province. Overall, the cold-hotspots are mainly located in the peripheral areas of the study area, while the central region is more of a transitional zone. These results indicate substantial regional heterogeneity in the spatial clustering patterns of bird richness across Southwest China.

### 3.2. Spatial Distribution Characteristics of Blue-Green Landscape Patterns

**Figure 4:**
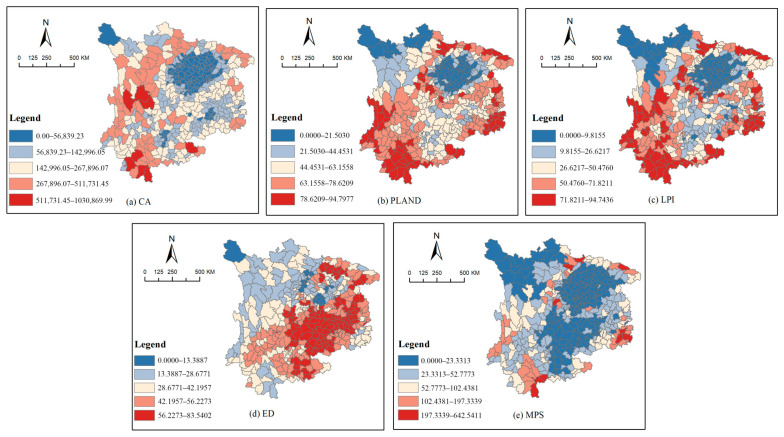
Spatial distribution of green space landscape pattern indices across Southwest China. (**a**) Class Area; (**b**) Percentage of Landscape; (**c**) Largest Patch Index; (**d**) Edge Density; (**e**) Mean Patch Size.

As shown in Figure 4, green-space landscape indices exhibited substantial spatial heterogeneity across Southwest China. CA generally showed a pattern of higher values in peripheral regions and lower values in central areas, with relatively large green-space patches concentrated in the western parts of the study area. PLAND and LPI displayed similar spatial distributions, indicating relatively strong landscape dominance and continuity of green spaces in western Yunnan Province. In contrast, ED values were higher in eastern regions and lower in northwestern areas, suggesting relatively greater fragmentation and edge effects in the eastern part of the study area. MPS values were generally higher in southwestern regions, reflecting comparatively larger and more connected green-space patches, whereas other regions exhibited relatively fragmented landscape structures overall. These patterns indicate pronounced regional differences in green-space configuration and connectivity across Southwest China.

**Figure 5:**
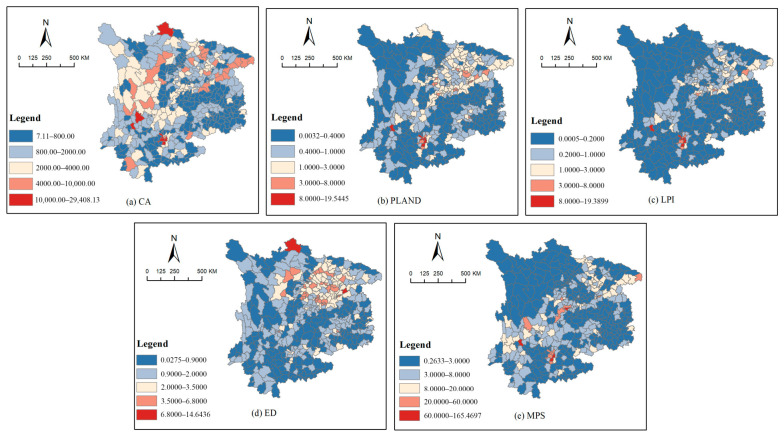
Spatial distribution of blue space landscape pattern indices across Southwest China. (**a**) Class Area; (**b**) Percentage of Landscape; (**c**) Largest Patch Index; (**d**) Edge Density; (**e**) Mean Patch Size.

As shown in Figure 5, blue-space landscape indices generally exhibited relatively low overall values across the study area, indicating the limited spatial extent of blue spaces in Southwest China. High CA values were mainly concentrated in western regions, while elevated ED values were primarily observed in northern areas, suggesting spatial heterogeneity in blue-space distribution and fragmentation. In contrast, PLAND, LPI, and MPS exhibited relatively scattered distributions with similar spatial patterns across the study area, whereas southern regions consistently showed relatively low values for these indicators. Overall, blue-space landscape patterns exhibited fragmented and spatially heterogeneous distributions, reflecting substantial regional differences in aquatic landscape structure throughout Southwest China.

### 3.3. The Driving Factors of Bird Richness

#### 3.3.1. SHAP Feature Importance and Beeswarm Plot Analysis

The optimized Random Forest model showed moderate predictive performance for bird richness. On the testing dataset, the model achieved an R^2^ value of 0.385 and an RMSE value of 66.86. These results indicate that the model captured part of the variability in bird richness while maintaining acceptable generalization ability for ecological data characterized by complex nonlinear relationships.

**Figure 6:**
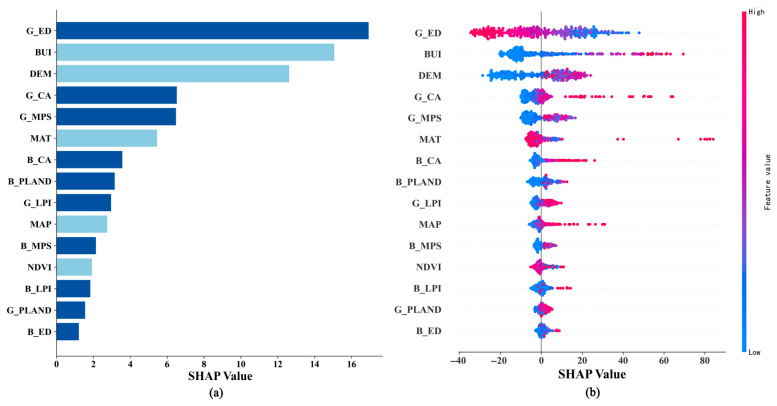
Factors Influencing Bird Richness. (**a**) Global Feature Importance Plot; (**b**) SHAP Beeswarm Plot. Note: Dark blue bars represent landscape pattern indicators; Light blue bars represent control variables.

The importance of global features (Figure 6a) indicates that the three most influential indicators affecting bird richness are G_ED and DEM, with G_ED exhibiting significantly greater feature importance than other factors. In contrast, the impact of green space indicators G_PLAND and G_LPI, as well as blue space indicators B_ED and B_LPI, is relatively minor.

The SHAP beeswarm plot (Figure 6b) reveals that for the ED value with the highest feature importance, higher green space edge density produces a more pronounced negative effect at feature points, while lower edge density yields a more pronounced positive effect, with overall results showing broad dispersion. BUI is primarily positively correlated with bird richness. Higher BUI values correspond to stronger positive SHAP values, which in turn have a positive effect on increasing bird richness. For DEM, higher values tended to exert positive associations. Unlike ED, DEM exerted the greatest influence on bird richness at moderate levels. G_CA, G_MPS and annual precipitation exhibited similar associations with bird richness, with high values typically exerting significant positive associations. Annual temperature showed unstable associations, with a small number of high values exerting substantial associations. Blue space variables CA and PLAND exhibit similar associations with bird richness, with higher values showing positive but modest associations. Other variables, such as NDVI, green space LPI, PLAND, blue space MPS, LPI, and ED, cluster near zero values, indicating negligible or no influence.

#### 3.3.2. SHAP Dependency Analysis

**Figure 7:**
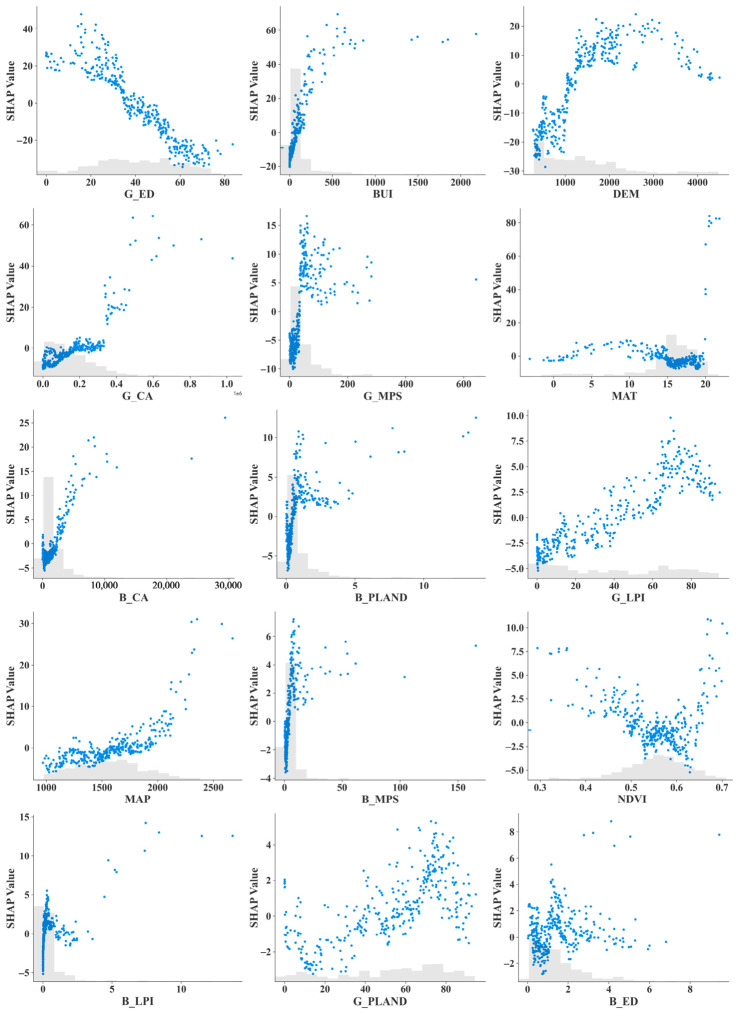
SHAP Dependency Plot. Note: The x-axis represents the values of explanatory variables, while the y-axis represents the corresponding SHAP values, indicating the direction and magnitude of each variable’s contribution to bird richness predictions. Positive SHAP values indicate positive associations with bird richness, whereas negative values indicate negative associations. The gray histograms in the background represent the density distribution of samples within different value ranges of each explanatory variable.

The SHAP Dependency Plot (Figure 7) visually illustrates the positive, negative, and nonlinear relationships between different feature variables and bird richness.

G_ED exhibits a highly pronounced near-linear negative relationship: as ED values increase, its negative impact on bird richness becomes more pronounced. There is a significant nonlinear positive correlation between BUI and bird richness. In the low range where BUI approaches 0, SHAP values are predominantly negative. As BUI increases, SHAP values turn from negative to positive and show a sharp overall upward trend. Once BUI reaches approximately 700, SHAP values show little fluctuation. DEM exhibits an inverted U-shaped trend in its impact on bird richness. Different elevations provide varying degrees of favorable conditions for bird richness. DEM begins exerting a positive influence around 1200 m, peaks at approximately 2500 m, and then gradually diminishes. Terrain in mid-elevation zones is often complex and less impacted by human activities, providing relatively secure habitats for birds. G_CA exerts its strongest negative associations with bird richness at values lower than 0.5 × 10^5^ ha, turning positive at approximately 0.2 × 10^6^ ha, with its influence increasing as CA values rise. Annual mean temperature showed a weak U-shaped association with bird richness, with SHAP values remaining relatively low between approximately 3 and 15 °C and increasing markedly above 20 °C. Annual mean precipitation showed an overall positive association with bird richness, with SHAP values increasing substantially when precipitation exceeded approximately 1750 mm. Compared with precipitation, annual mean temperature exhibited a stronger association with bird richness. The nonlinear relationship observed at higher temperatures may be related to increasing environmental heterogeneity and human activities in warmer regions. Higher precipitation levels may also be associated with more humid and vegetated environments, which are generally linked to greater habitat availability and food resources for birds. NDVI exhibited a V-shaped association with bird richness, with SHAP values decreasing initially and becoming negative at approximately 0.5, followed by an increasing trend that turned positive again above approximately 0.65. For blue space indicators CA, LPI, PLAND, and MPS, a near-universal positive relationship emerges: increasing values yield greater positive associations with bird richness, with influence stabilizing beyond specific thresholds. Conversely, G_MPS and B_ED exhibit unstable associations with bird richness. G_PLAND and G_LPI exhibited similar nonlinear relationships with bird richness, characterized by increasing SHAP values at lower levels followed by declines after reaching threshold values.

## 4. Discussion

### 4.1. Analysis of Blue-Green Space Landscape Characteristics Primarily Influencing Bird Richness

Bird aggregation patterns typically reflect the spatial distribution of birds within a region and their relationship with ecological and environmental factors. High values and low values, respectively, indicate high concentration or low dispersion of birds within the area, revealing certain ecological and environmental characteristics.

High-value bird aggregation sites are located in northern Sichuan Province and western Yunnan Province, with landscape pattern characteristics revealing complementary interactions between green spaces and blue spaces. First, regarding green space, the region possesses extensive total green area with low fragmentation and good patch connectivity. It maintains large core areas with minimal edge effects, potentially providing relatively suitable habitat conditions for birds [15]. Simultaneously, highly connected patches potentially support habitat connectivity and movement among bird populations, thereby contributing to relatively stable habitat conditions and higher bird richness [40]. Second, regarding blue space, the region features extensive water bodies with relatively good connectivity but high fragmentation. Although scattered water patches may increase risks to some extent, the relatively large total area and connectivity still potentially support movement and habitat use by waterbirds between different water patches, thereby alleviating local resource competition [41]. Overall, the continuity and connectivity of green space ensure avian stability within terrestrial ecosystems, while the fragmentation of blue space increases aquatic diversity and may increase landscape heterogeneity. This combined landscape pattern is associated with the relatively high bird richness observed in northern Sichuan and western Yunnan.

The low-value areas for bird aggregation are located in eastern Sichuan Province and central Guizhou Province. Their landscape pattern characteristics reveal limitations in the extent, connectivity, and fragmentation of blue-green spaces, which negatively impact bird aggregation. First, regarding green spaces, the total area and proportion of green spaces in this region are both low, with poor patch connectivity and relatively high fragmentation. Green patches are scattered and highly isolated, with small core areas and significantly increased edge effects, exposing more regions to external disturbances. In contrast, edge zones typically experience stronger sunlight, higher wind speeds, reduced humidity, and greater variability in plant community structure and insect resource composition, posing potential impacts on biodiversity [42]. Additionally, human activities, noise, and light pollution are often more prevalent in edge zones, potentially increasing environmental disturbance and reducing habitat suitability for some bird species [43]. Low connectivity hinders avian migration between patches, which may limit connectivity between habitats and thereby reduce the ecological value of bird aggregations. [44]. Secondly, regarding blue space, this area exhibits poor water connectivity and fragmented patch distribution. Although water bodies are associated with aquatic habitat heterogeneity, the isolated and low-connectivity nature of these patches may reduce functional connectivity among waterbird habitats. The loss of functional connectivity within migration networks may hinder energy replenishment during migration and compromise the maintenance of optimal body reserves for reproduction [40]. Overall, the landscape pattern characteristics of blue and green spaces in this region significantly constrain bird aggregation. Green areas with low coverage, high fragmentation, and low connectivity may reduce the availability of relatively stable habitats and movement pathways for terrestrial birds. Although water bodies are abundant in total volume, their fragmented patches and insufficient connectivity result in low foraging efficiency and limited habitat utilization for waterbirds. This landscape configuration is associated with relatively low observed bird richness in eastern Sichuan and central Guizhou.

Heterogeneous variations in landscape patterns can exert direct or indirect influences on bird communities through differences in habitat availability, connectivity, environmental heterogeneity, and human disturbance [26]. At the regional scale, the spatial aggregation patterns observed in Southwest China mainly reflect differences in blue-green landscape configurations and habitat conditions associated with bird richness. Areas with relatively high bird richness may support different assemblages of species under varying environmental contexts, including both habitat-sensitive and disturbance-tolerant species. Therefore, bird richness in this study should primarily be interpreted as an indicator of regional biodiversity patterns and spatial ecological heterogeneity, rather than a direct measure of ecological integrity alone. Based on these regional patterns, the following analysis further discusses the landscape characteristics commonly associated with bird richness in Southwest China.

Green spaces provide habitat and food resources, and differences in internal structure may support different avian assemblages with varying feeding habits and ecological requirements [45]. Research indicates that edge density within green spaces exerts the strongest influence on bird richness. Under conditions of low green space fragmentation, this effect is significant, whereas moderate to high fragmentation levels exert a negative impact on bird richness. Green landscapes in the southwest region predominantly exhibit discontinuous, patchy patterns. On the one hand, high fragmentation manifests as the overall division of green spaces into small patches, resulting in a lack of core habitats and the near-disappearance of internal habitat space. This may reduce habitat heterogeneity and the availability of food and nesting resources for some bird species [46]. This increases birds’ dependence on surrounding environments; if these areas are disturbed by human activities, bird richness is directly affected [47]. On the other hand, green space fragmentation rarely acts alone; it often interacts with roads, highways, and railways, indirectly amplifying the adverse effects of human disturbance on bird communities [26]. Furthermore, the total area of green space significantly influences bird richness in a positive manner—larger areas correlate with higher richness, a finding confirmed by existing research [18]. On the one hand, larger green spaces maintain more complex and stable habitat conditions. These potentially provide more diverse habitat conditions and resources that are associated with higher bird richness. Smaller green spaces, however, often only accommodate more generalist bird species, resulting in lower diversity [23]. On the other hand, at the urban scale, the total green space area also determines the city’s overall habitat availability. If the greening rate is low, even the presence of a few large green spaces may still hinder birds’ ability to sustain migration and dispersal processes. Conversely, when the total green space area is sufficient and distributed appropriately, different bird species can maintain a balanced state through migration between spatial green corridors and green space patches [27].

Blue spaces provide important aquatic habitat conditions for birds. Areas such as water margins, wetlands, and shallow water zones serve as ideal sites for bird foraging and breeding [48]. The geographical characteristics of blue spaces support rich biodiversity, sustaining bird populations’ survival and reproduction [49]. In southwestern China, complex mountainous and plateau landscapes create highly heterogeneous distributions of water bodies, with natural lakes and artificial reservoirs representing major aquatic habitats. Under such environmental conditions, moderate blue-space area and coverage may contribute to greater habitat heterogeneity by increasing the availability of aquatic–terrestrial transitional zones, which are often associated with higher bird richness. These ecotone environments may provide a variety of food resources, shelter conditions, and potential nesting opportunities for different bird species [50]. As blue-space area and landscape proportion continue to increase, however, their ecological associations with bird richness may gradually weaken. One possible explanation is that aquatic habitat availability reaches a saturation state beyond certain thresholds, after which additional increases in water area contribute relatively limited gains in habitat diversity and ecological resources. At the same time, excessively dominant water bodies may simplify the surrounding landscape composition and reduce the mosaic structure formed by aquatic and terrestrial habitats. Such homogenization may limit the coexistence of bird species with different habitat preferences [51,52]. Therefore, maintaining a balanced proportion of blue spaces within heterogeneous landscapes may be more beneficial for sustaining bird richness than simply increasing water coverage.

### 4.2. Recommendations for Bird Conservation and Strategies for Improving Landscape Features in Southwest China

This study elucidates the complex dynamics between landscape characteristics and biodiversity. Based on findings regarding the relationship between bird richness and landscape pattern features, several recommendations are proposed to enhance bird conservation and ecological environment improvement: (1) Given the strong association between green edge density and bird richness, optimizing the morphology and configuration of green patches is a key strategy for enhancing bird habitat quality. Research indicates that small habitat patches generate landscape heterogeneity. A key aspect of this heterogeneity is increased habitat edges [53]. Merging adjacent small patches or establishing “ecological buffers” can reduce edge length per unit area, thereby lowering ED values. For instance, urban parks and mountain forest belts should prioritize internal expansion into contiguous areas rather than adding narrow, elongated edge green spaces. This approach increases the proportion of core areas, potentially providing more stable habitat conditions for forest-dwelling birds [15]. In these areas, establishing interconnected park systems and ecological corridors between green spaces can further reduce habitat fragmentation and support wildlife migration within fragmented urban landscapes. Such strategies help enhance the ecological functions of urban green spaces and strengthen the capacity of urban ecosystems to support biodiversity conservation. Additionally, establishing a gradient structure from “core-transition-edge” through planting shrubs, mixed tree belts, or wetland plants can buffer the impact of external disturbances on birds [54]. (2) Increasing total green space area is associated with relatively higher bird richness and abundance. However, converting “area” into effective habitat requires attention to patch size, core areas, and other factors. Research indicates that prioritizing the retention or creation of large patches is an effective approach for increasing green space area. Forest birds or breeding birds sensitive to the area typically require at least several dozen hectares of contiguous habitat to maintain high breeding success rates. Studies suggest minimum thresholds for certain species range around 15–20 ha [55]. Second, ensure core areas are located away from edges, ideally within primary forest blocks or parks. Alternatively, enhance effective core depth by thickening forest strips and reducing human disturbance [56]. (3) A significant positive correlation exists between water body size and bird richness. Therefore, while strengthening protection of existing water bodies to prevent filling, pollution, and overdevelopment—ensuring no reduction in water area—urban and regional planning should also improve the spatial continuity of rivers, wetlands, and reservoirs. Increasing water area through wetland restoration and ecological water system construction may provide more stable habitats and foraging environments for birds [57]. Additionally, research indicates that the species richness and paired abundance of a single large wetland can be comparable to those of several small wetlands. However, when resources are limited at the regional scale, particularly in landscapes with existing large natural wetlands, constructing multiple small wetlands should be prioritized over a few large ones [19].

Overall, these findings suggest that biodiversity-oriented blue-green space planning can help improve habitat quality and ecological connectivity in rapidly urbanizing regions of Southwest China. Integrating connected parks, ecological corridors, and continuous river–wetland systems into urban planning may reduce habitat fragmentation and better support bird diversity and wildlife survival in complex urban landscapes. By enhancing the ecological functionality and spatial integrity of urban blue-green infrastructure, these strategies may also contribute to more resilient and ecologically sustainable urban development.

## 5. Conclusions

This study investigates the association patterns of blue-green spaces on bird richness across counties in Southwest China, utilizing six landscape pattern indicators and three control indicators. By integrating random forest models with SHAP analysis, we assess the importance of landscape features and their nonlinear relationships with bird richness. Key findings include: (1) High-value spatial clusters of bird richness were concentrated in northern Sichuan and western Yunnan, while low-value clusters were found in eastern Sichuan and central Guizhou. (2) Significant variations in green space landscape patterns existed across counties, with CA, PLAND, and LPI exhibiting similar distribution patterns throughout the study area. Blue space landscape patterns showed relatively minor inter-county differences. (3) Global feature importance results indicated nonlinear relationships between various landscape characteristics and bird richness in the Southwest region. The three most influential indicators for bird richness were G_ED, BUI, and DEM, with G_ED exhibiting significantly greater feature importance than the other factors. In summary, this study highlights the differentiated associations of blue and green landscape configurations with bird richness, providing crucial scientific evidence and practical recommendations for advancing ornithological research and achieving ecological balance.

However, the study still has certain limitations: (1) The citizen-science bird observation data may contain regional differences in observation intensity, which could influence the spatial patterns of bird richness. (2) Bird richness was analyzed as an overall community indicator without distinguishing among functional groups or migratory types, which may limit ecological interpretation. (3) Although Moran’s I indicated significant spatial clustering, spatial autocorrelation was not explicitly incorporated into the Random Forest framework, and some ecological associations may still partially reflect underlying spatial dependence. (4) This study focused primarily on taxonomic diversity and did not include functional diversity indices such as FRic, FDis, or CWM.

## Figures and Tables

**Table 1:** Data Used in This Study.

Data Type	Data Source	Data Description
Bird data	China Bird Report Center (http://www.birdreport.cn (accessed on 10 November 2025))	Statistical bird richness data
Land use data	Annual China Land Cover Dataset (https://zenodo.org/records/15853565 (accessed on 15 November 2025))	Extract data on blue-green spaces such as forests, shrublands, water bodies, and wetlands
DEM	Geospatial Data Cloud (https://www.gscloud.cn (accessed on 15 November 2025))	Derived from the original elevation data of SRTMDEM
Precipitation data	ERA5-Land Daily Aggregated	Calculate the average annual precipitation
Temperature data	ERA5-Land Daily Aggregated	Calculate the annual average temperature
NDVI	MOD13A3	Characterize vegetation productivity
BUI	GHSL Built-up Surface (GHS-BUILT-S)	Characterize urbanization intensity and human disturbance

**Table 2:** Definitions and calculation formulas of selected landscape pattern indices for blue-green spaces.

Index	Uint	Formula	Formula Explanation
CA	ha	$\sum_{j=1}^{n}a_{ij}$	$a_{ij}$: Area of the *j*th patch in landscape type *i* *n*: Number of plaques of this type
ED	m/ha	$\frac{\sum_{j=1}^{n}e_{ij}}{A}\times 10000$	$e_{ij}$: Edge length of the *j*th patch in landscape type *i* *A*: The total area of the entire landscape
LPI	%	$\frac{max(a_{ij})}{A}$	$max(a_{ij})$: Maximum plaque area *A*: The total area of the entire landscape
PLAND	%	$\frac{CA}{A}\times 100$	*A*: The total area of the entire landscape
MPS	ha	$\frac{CA}{n}$	*n*: Number of plaques of this type

## Data Availability

The original data presented in the study are openly available in FigShare at https://doi.org/10.6084/m9.figshare.32146708.

## Abbreviations

The following abbreviations are used in this manuscript:

RF	Random Forest
SHAP	Shapley
CA	Class Area
ED	Edge Density
LPI	Largest Patch Index
PLAND	Percentage of Landscape
MPS	Mean Patch Size
MAT	Mean Annual Temperature
MAP	Mean Annual Precipitation
NDVI	Normalized Difference Vegetation Index
BUI	Built-up Intensity
